# 3D motion analysis dataset of healthy young adult volunteers walking and running on overground and treadmill

**DOI:** 10.1038/s41597-024-03420-y

**Published:** 2024-05-30

**Authors:** Louis Riglet, Corentin Delphin, Lauranne Claquesin, Baptiste Orliac, Paul Ornetti, Davy Laroche, Mathieu Gueugnon

**Affiliations:** 1 INSERM, CIC 1432, Module Plurithématique, Plateforme d’Investigation Technologique, 21000 Dijon, France; 2 CHU Dijon-Bourgogne, Centre d’Investigation Clinique, Module Plurithématique, Plateforme d’Investigation Technologique, 21000 Dijon, France; 3 INSERM, UMR1093-CAPS, Univ. Bourgogne Franche-Comté, UB, 21000 Dijon, France; 4 Rheumatology department, CHU Dijon-Bourgogne, 21000 Dijon, France; 5 Collaborative Research Network STARTER, Innovative Strategies and Artificial Intelligence for Motor Function Rehabilitation and Autonomy Preservation, 21000 Dijon, France

**Keywords:** Biomedical engineering, Medical research

## Abstract

Used on clinical and sportive context, three-dimensional motion analysis is considered as the gold standard in the biomechanics field. The proposed dataset has been established on 30 asymptomatic young participants. Volunteers were asked to walk at slow, comfortable and fast speeds, and to run at comfortable and fast speeds on overground and treadmill using shoes. Three dimensional trajectories of 63 reflective markers, 3D ground reaction forces and moments were simultaneously recorded. A total of 4840 and 18159 gait cycles were measured for overground and treadmill walking, respectively. Additionally, 2931 and 18945 cycles were measured for overground and treadmill running, respectively. The dataset is presented in C3D and CSV files either in raw or pre-processed format. The aim of this dataset is to provide a complete set of data that will help for the gait characterization during clinical gait analysis and in a sportive context. This data could be used for the creation of a baseline database for clinical purposes to research activities exploring the gait and the run.

## Background & Summary

Three-dimensional motion analysis system is broadly used to capture human movements, especially during walking and running who is considered as the main conditions of human locomotion^1^. This kind of system is considered as the most reliable and most accurate measurements of movement^2–4^ especially for spatiotemporal, kinematic and dynamic parameters used to assess the gait pattern of the individual (i.e. walking or running). In a clinical context, motion analysis is used for diagnostic and evaluation of treatment for specific neurological, muscular or orthopaedics pathology by quantify the deviations from normal gait^5,6^. In the same context, running motion analysis can be used as clinical tools for diagnostic and evaluation of treatment of sports injuries and could improve measurement performance, notably in sports population^7,8^.

Human data set are more and more frequently published and examples of similar gait analysis dataset are already available on pathologic patients^9,10^ and healthy participants^9,11–14^. Notably, Schreiber and Moissenet^11^ published a dataset of adults healthy and injury-free but focusing only on five walking speeds. Additionally, Fukuchi *et al*.^15^ presented a complete dataset of overground and treadmill walking kinematics and kinetics in healthy individuals but focusing only on the lower limb. Moreover, Mei *et al*.^14^ published a database of joint angles, moments, and forces of the lower extremity from distance running at a submaximal speed in recreational runners. Despite these data availabilities, to our knowledge, no study had provided both run and walk dataset on the same participants at different speeds and conditions (treadmill and overground) and in large number of participants. Because speed and environment (overground or treadmill) were found to be parameters modifying individual walking and running^15–17^, it appears to be a lack of available data. Notably, in a clinical context, control group are usually limited by the number of participants and focus on comfortable walking tasks. However, this speed is commonly faster than that of individuals in the pathological population^18^. Such experimental dataset could help scientists to describe how the gait parameters are modified, and could be a reference to compare with pathological or sport population. From another perspective, in order to improve either biomechanics simulation or machine learning tools such kind of data would help to feed algorithms and then to assess their external validity. For these reasons, the dataset cover broad scientific fields and could be used for multiple purposes from the analysis of pathological motion^19^ to the simulation of walking and running biomechanics^20,21^ either in fundamental or applied sciences. For instance, a small part of this present dataset has already been used in a validity and test-retest reliability study. For walking task, Riglet *et al*.^22^ used the 3D lower limb markers trajectories to confirm the relevant measurement of the spatiotemporal gait parameters from wearable insoles. However, all data (3D full-body markers trajectories, kinematics, kinetics, etc.) has not yet been used and seems relevant to the entire scientific community. Moreover, motion analysis requires a long operation time, dedicated space requirement, technical expertise requirement and high cost^2^. Thus, data sharing seems essential in the development of scientific research to improve representation of human variability, improve analysis strategies, etc^23,24^.

The primary objective of this study was to provide a dataset established on 30 asymptomatic healthy participants aged between 21 and 41 years. They were asked to walk on overground and motorized treadmill at three different walking speeds (slow, comfortable and fast) with identical shoes during two different sessions. Moreover, they were also asked to run at comfortable and fast speeds on overground and treadmill. Three dimensional trajectories of 63 cutaneous reflective markers, based on the predominant biomechanical marker set (Conventional Gait Model^25^), and 3D ground reaction forces and moments were recorded (overground only). A total of 4840 and 18159 gait cycles were measured for overground and treadmill walking, respectively. For running task, 2931 and 18945 cycles were measured on overground and treadmill, respectively. In order to allow the scientific community to be able to process data as they wish, the dataset was presented in C3D and CSV files with and without post-processing.

## Methods

### Participants

Thirty healthy participants (16 men, 14 women, 28.0 ± 5.6 years, 1.73 ± 0.09 m, 68.2 ± 11.1 kg) were recruited from September 2021 to January 2022 in the Dijon University Hospital (France). The inclusion criteria were: healthy subjects, older than 18 years old, who was able to understand simple orders and instructions for locomotion and who lived within a maximum radius of 50 km from the investigation site. The major exclusion criteria were persons who were not affiliated to a national health insurance, subject to a legal protection measure, unable to express consent, presented a disarticulated hip, diseases or disabilities that have an impact on walking or with comorbidities that potentially affect the gait pattern. All participants included in this study provided their informed oral consent in accordance with the French law. The study protocol was developed in compliance with the Declaration of Helsinki and the Good Clinical Practice. It was approved by the ethics committee (CPP Ile-de-France X, n°RCB: 2021-A01058-33) and authorized by the French National Agency for Drug Safety. This study was referenced on Clinical Trial registration (NCT05104645).

### Procedure

**Fig. 1:**
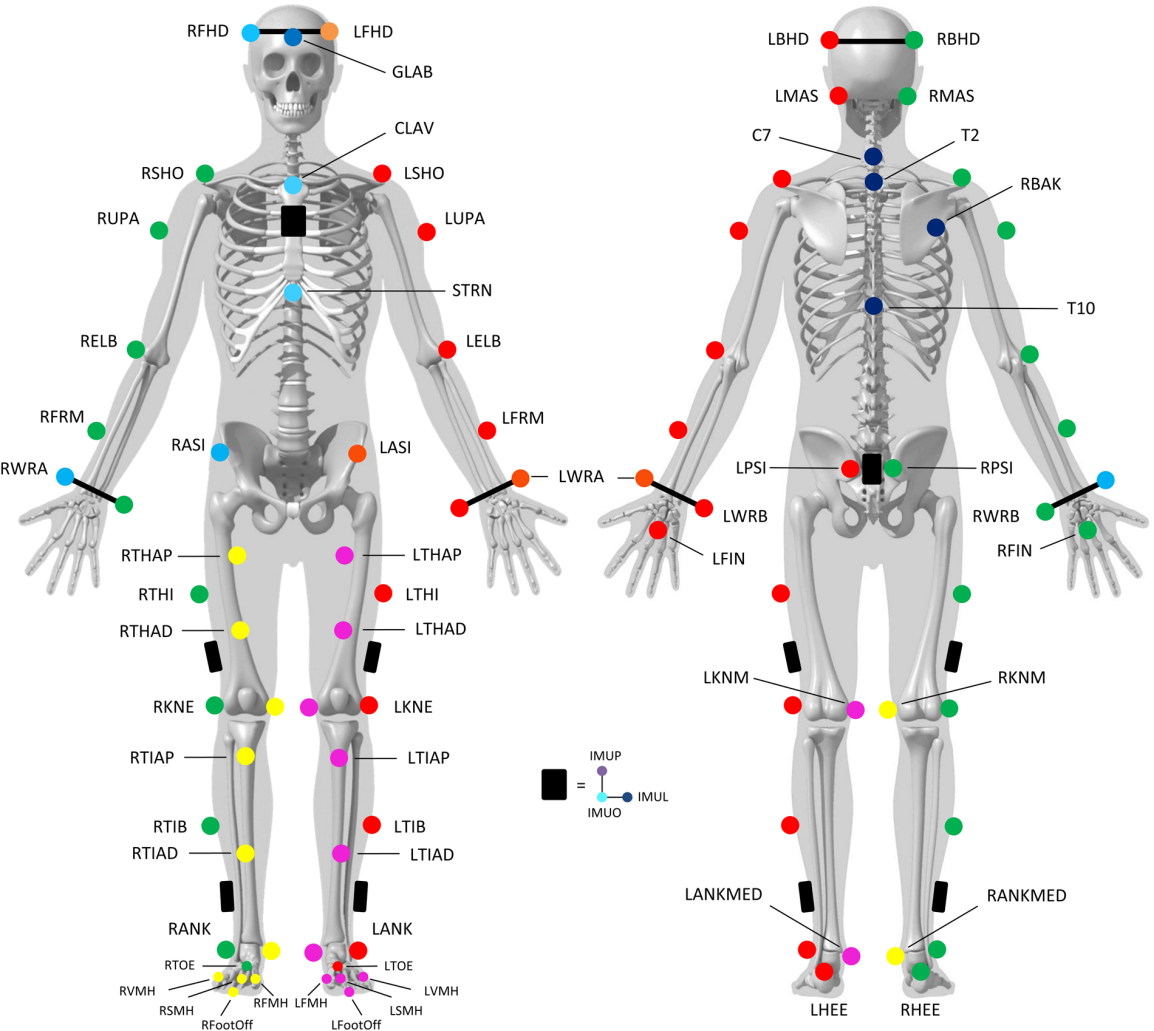
Position of the markers on the anatomical landmarks following the Conventional Gait Model (version 2.5).

**Fig. 2:**
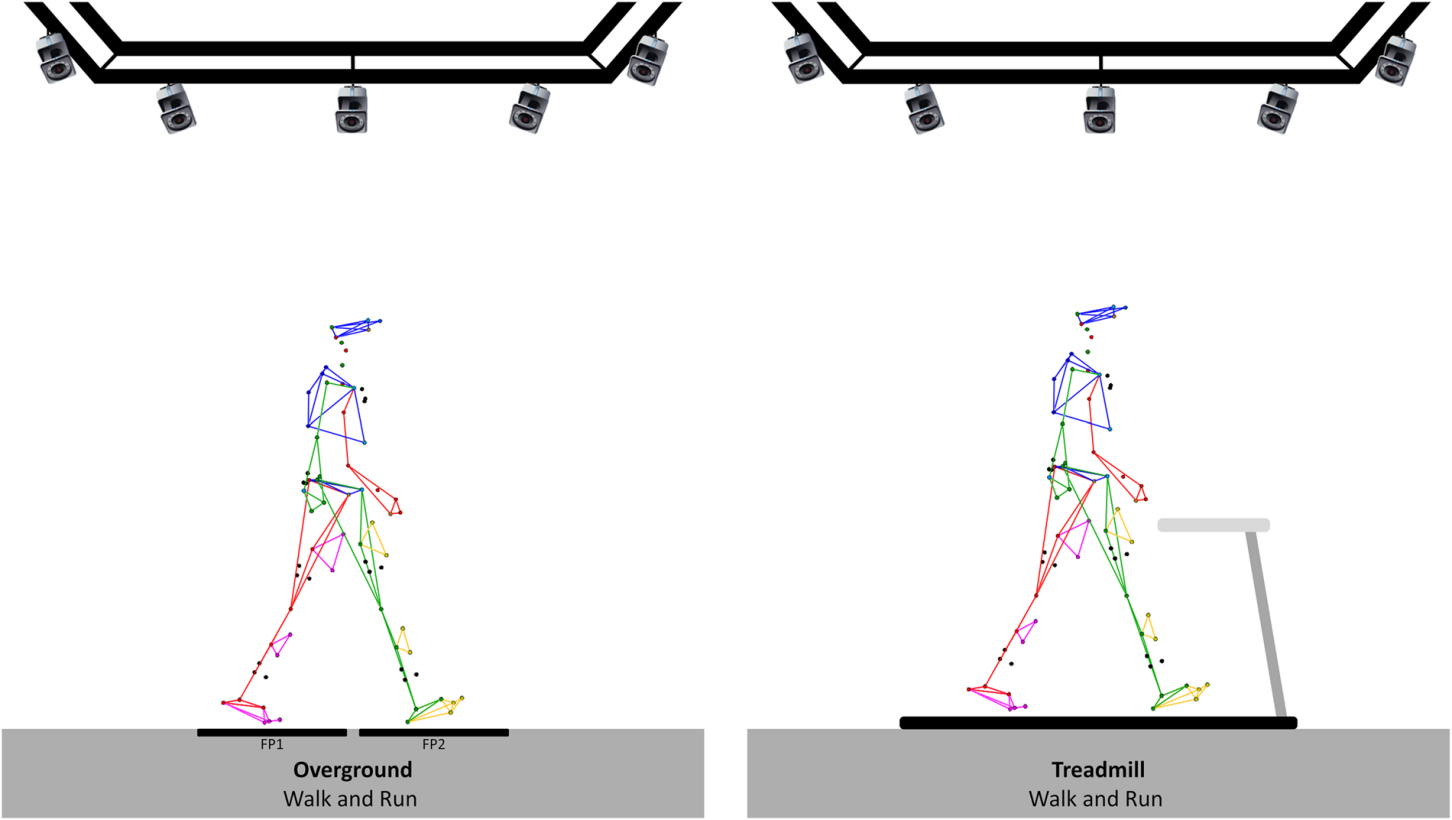
Illustration of walking and running trials. FP = Force plate.

For each healthy participant, the entire data collection was recorded in two sessions which lasted approximately 2 hours. The following procedure was adopted for the first session:

1. Consent information to the participant: An investigator of the study introduced the laboratory, outlined the main objective of the study, and explained the procedure and how to conduct the two sessions.
2. Medical interview: The interview aims to gather demographics (age, sex, height and weight) and to verify the inclusion/exclusion criteria.
3. Calibration of the systems: The calibration was performed following the instructions available in the manufacturer’s documentation, including the definition of the inertial coordinate system, the dynamic calibration of the cameras, and the zeroing of forceplates.
4. Preparation of the participant: The participant was asked to change clothes to tight-fitting clothes or underwear and wear walking shoes provided for the experiment (Ekiden One, Kalenji®). The operator also collected participants’ anthropometric information needed for the markerset. All participants were equipped with reflective cutaneous markers positioned following the Conventional Gait Model^26^, as shown in Fig. 1.
5. Calibration file/static record: The participant was standing upright with lower limbs outstretched, upper limbs bent at 90° with palms facing the floor, right head with straight eyes. Five seconds without any movement were recorded. A new record was performed if any marker was missing or misplaced regarding the Conventional Gait model. This file is named “Calibration” in the dataset and included in each volunteer folder.
6. Walking (Fig. 2):

- Overground walking trials: The participant was asked to walk, with shoes, along a line drawn on the ground (~10 m) at their spontaneous walking speed, then slow and fast. No directive was given about the force plates to avoid a conscious adaptation of the walk. A minimum of 3 platform steps per leg were recorded for each condition.
- Treadmill walking trials: The participant was asked to walk with shoes, on a motorized treadmill at slow speed, comfortable and fast. The comfortable walking speed was selected by the volunteer after a 2-minute familiarization trial. The slow speed was then calculated by removing 1 km/h from the comfortable speed and the fast speed was calculated by adding 2 km/h. Each speed condition was performed for 2 minutes, followed by 1 minute of recovery.

7. Running trials (Fig. 2):

- Overground running trials: The participant was asked to run with shoes provided for the experiment, along a straight line drawn on the ground (~6 m) while running. The size of the room makes it possible to start the race and finish it with sufficient space (at least 5 meters on either side) at a comfortable then fast speed. A minimum of 3 platform steps per leg were recorded for each condition.
- Treadmill running trials: The participant was asked to run, with shoes provided for experimentation on a motorized treadmill at comfortable and fast speeds. The comfortable running speed was selected as treadmill walking trials, and fast speed was calculated with the same way as in walking trials. Each speed condition was performed for 2 minutes, followed by 1 minute of recovery.

8. Session ending: All markers and electrodes were removed. Additional explanations about the records were given to the participants while showing some videos and 3D animations.

The second session was performed 7 days after the first one and was composed by the step three to eight. Visits were performed at the same day of the week and at similar time slots to restrict gait day-fluctuations^27^.

For each session, a complete list of volunteers’ metadata is available and included: ID of volunteers, demographic parameters (age, sex, height, weight) and anthropometric parameters related to the Conventional Gait Model.

### Records

Using 18 optoelectronic cameras operating at a sampling rate of 100 Hz (11 VERO and 7 MX-T10 cameras, Vicon System®, Oxford, UK; 100 Hz) and Nexus software (2.12.1 version), the three-dimensional (3D) trajectories of a set of 63 cutaneous reflective markers was tracked (Fig. 1). The marker set was defined following the Conventional Gait Model (version 2.5) full-body markers set^26,28^. One marker was added (R/L ToeOff) at the extremity of the shoes, close to the hallux. IMU systems equipped with 3 markers were also fixed on the participants (data from IMU are not available - Property of an external company). A full description of each marker is reported in Table 1. Two force plates were embedded in the floor to record ground reaction forces (AMTI®, USA; 1000 Hz) and one numerical camera was positioned in the sagittal plane to check platform steps (piA640-210gc, Basler Pilot®, USA; 50 Hz). A description of force plate data is reported in Table 2. All these systems were synchronized using Vicon Giganet® hardware and Nexus^®^ software (Vicon System®, Oxford, UK).

**Table 1:** Real marker trajectories of the Conventional Gait Model stored in post-processed files.

Labels	Description	Position on participant
(L/R)FHD	Left/right front head	Left/right temple
(L/R)BHD	Left/right back head	Left/right back of head
(L/R)MAS	Left/right mastoid process	Behind the left/right earlobe
GLAB	Forehead middle	On the headband
C7	7^th^ cervical vertebra	On the spinous process of the 7^th^ cervical vertebra
T2	2^nd^ thoracic vertebra	On the spinous process of the 2^nd^ thoracic vertebra
T10	10^th^ thoracic vertebra	On the spinous process of the 10^th^ thoracic vertebra
CLAV	Clavicle	On the jugular notch where the clavicles meet the sternum
STRN	Sternum	On the xiphoid process of the sternum
RBAK	Right back	On the middle of the right scapula (not symmetrical on the left)
(L/R)SHO	Left/right shoulder	On the left/right acromio-clavicular joint
(L/R)UPA	Left/right arm	Upper 1/3 of the lateral aspect of the left/right arm
(L/R)ELB	Left/right elbow	On the left/right lateral epicondyle
(L/R)FRM	Left/right forearm	Lower 1/3 of the lateral aspect of the left/right forearm
(L/R)WRA	Left/right wrist marker A	Left/right radius styloid process
(L/R)WRB	Left/right wrist marker B	Left/right ulnar styloid
(L/R)FIN	Left/right finger	Middle of the back of the left/right hand
(L/R)ASI	Left/right ASIS	Left/right anterior superior iliac spine
(L/R)PSI	Left/right PSIS	Left/right posterior superior iliac spine
(L/R)THI	Left/right thigh	Halfway up the lateral left/right thigh
(L/R)THAP	Left/right thigh	Proximal 1/3 of the left/right thigh (anterior)
(L/R)THAD	Left/right thigh	Distal 1/3 of the left/right thigh (anterior)
(L/R)KNE	Left/right knee	On the flexion-extension axis of the left/right knee (lateral epicondyle)
(L/R)KNM	Left/right knee	On the flexion-extension axis of the left/right knee (medial epicondyle)
(L/R)TIB	Left/right tibia	Halfway up the lateral left/right leg
(L/R)TIAP	Left/right tibia	2 cm below the left/right tibial tuberosity
(L/R)TIAD	Left/right tibia	Halfway up the left/right leg (on the tibial crest)
(L/R)ANK	Left/right ankle	Left/right lateral tibial malleolus
(L/R)MED	Left/right ankle	Left/right medial tibial malleolus
(L/R)HEE	Left/right heel	Most prominent part of the posterior calcaneus
(L/R)TOE	Left/right toe	Metatarsocuneiform joint of the left/right 2^nd^ toe
(L/R)FMH	Left/right 1^st^ toe	Proximal metatarsophalangeal joint of the left/right 1^st^ toe
(L/R)VMH	Left/right 5^th^ toe	Proximal metatarsophalangeal joint of the left/right 5^th^ toe
(L/R)SMH	Left/right 2^nd^ toe	Proximal metatarsophalangeal joint of the left/right 2^nd^ toe
(L/R)FootOff	Left/right shoe	Extremity of the left/right shoe, close to the hallux
TrunkIMUO	IMU on trunk	On the lower left corner of the IMU fixed on the trunk
TrunkIMUP	IMU on trunk	On the upper left corner of the IMU fixed on the trunk
TrunkIMUL	IMU on trunk	On the lower right corner of the IMU fixed on the trunk
PelvisIMUO	IMU on pelvis	On the lower left corner of the IMU fixed on the pelvis
PelvisIMUP	IMU on pelvis	On the upper left corner of the IMU fixed on the pelvis
PelvisIMUL	IMU on pelvis	On the lower right corner of the IMU fixed on the pelvis
LFemurIMUO	Left IMU on femur	On the lower right corner of the IMU fixed on the left femur
LFemurIMUP	Left IMU on femur	On the upper right corner of the IMU fixed on the left femur
LFemurIMUL	Left IMU on femur	On the lower left corner of the IMU fixed on the left femur
LTibiaIMUO	Left IMU on tibia	On the lower right corner of the IMU fixed on the left tibia
LTibiaIMUP	Left IMU on tibia	On the upper right corner of the IMU fixed on the left tibia
LTibiaIMUL	Left IMU on tibia	On the lower left corner of the IMU fixed on the left tibia
RFemurIMUO	Right IMU on femur	On the lower left corner of the IMU fixed on the right femur
RFemurIMUP	Right IMU on femur	On the upper left corner of the IMU fixed on the right femur
RFemurIMUL	Right IMU on femur	On the lower right corner of the IMU fixed on the right femur
RTibiaIMUO	Right IMU on tibia	On the lower left corner of the IMU fixed on the right tibia
RTibiaIMUP	Right IMU on tibia	On the upper left corner of the IMU fixed on the right tibia
RTibiaIMUL	Right IMU on tibia	On the lower right corner of the IMU fixed on the right tibia

Dimensions of trajectories are 3 × N. Units are mm.

**Table 2:** Forceplates data stored in post-processed files.

Labels	Component	Units	Description
ForcePlate1	Force	(N, N, N, ms)	3D ground reaction Force (Fx1, Fy1, Fz1)
	Moment	(N.mm, N.mm, N.mm, ms)	3D ground reaction Moment (Mx1, My1, Mz1)
	Position	(mm,mm, mm, ms)	3D ground reaction Position (X1, Y1, Z1)
ForcePlate2	Force	(N, N, N, ms)	3D ground reaction Force (Fx2, Fy2, Fz2)
	Moment	(N.mm, N.mm, N.mm, ms)	3D ground reaction Moment (Mx2, My2, Mz2)
	Position	(mm, mm, mm, ms)	3D ground reaction Position (X2, Y2, Z2)

### Data processing

Two kinds of files will be available in the dataset. First, raw data files, stored in a binary c3d and CSV files, include any point trajectories (unlabelled names) and forces without any post-processing. Secondly, post processed files, stored in a c3d and CSV files, include the labelling of the marker trajectories and computation of the dynamics performed using the Vicon Nexus software (2.12.1 version). All details are presented in Tables 3, 4. Marker trajectories were interpolated with Woltring polynomial and then filtered with a low pass zero phase shift Butterworth filter with a respective cut off frequency of 6 Hz. Defined as virtual markers, landmark segments, joint centers, body center of mass (CoM) and ground reaction forces (GRF) normalized by the bodyweight were computed following the Conventional Gait Model using the Vicon Nexus® software (version 2.12.1)^25^. A full description of each virtual marker is reported in Table 3. Additionally, joint kinematics and kinetics were calculated based on the description of Baker *et al*.^25^ and are presented in Table 4. Then, the gait events (‘foot strike’ and ‘foot off’) were then computed using a method proposed by O’Connor *et al*.^29^ based on foot speed algorithm (using the virtual origin of the foot “FOOTO”). Gait cycle was defined using two successive foot strike of the same leg. The full contact of the left or right foot on the force platforms was defined by an experimental operator and confirmed using the numerical camera. Finally, post-processed measurements were cropped to obtain two files for each back and forth. Then, they were stored in a c3d and CSV file and renamed incrementally.

**Table 3:** Virtual marker trajectories of the Conventional Gait Model stored in post-processed files.

Virtual Marker	Description	Segment Coordinate System
HEADO	Head	Segment Origin
HEADA		Anterior axis
HEADP		Proximal axis
HEADL		Lateral axis
THORAXO	Thorax	Segment Origin
THORAXA		Anterior axis
THORAXP		Proximal axis
THORAXL		Lateral axis
PELVISO	Pelvis	Segment Origin
PELVISA		Anterior axis
PELVISP		Proximal axis
PELVISL		Lateral axis
(L/R)UPPERARMO	Left/right Humerus	Segment Origin
(L/R)UPPERARMA		Anterior axis
(L/R)UPPERARMP		Proximal axis
(L/R)UPPERARML		Lateral axis
(L/R)FOREARMO	Left/right Radius	Segment Origin
(L/R)FOREARMA		Anterior axis
(L/R)FOREARMP		Proximal axis
(L/R)FOREARML		Lateral axis
(L/R)HANDO = (L/R)HO	Left/right Hand	Segment Origin
(L/R)HANDA		Anterior axis
(L/R)HANDP		Proximal axis
(L/R)HANDL		Lateral axis
(L/R)FEMURO	Left/right Femur	Segment Origin
(L/R)FEMURA		Anterior axis
(L/R)FEMURP		Proximal axis
(L/R)FEMURL		Lateral axis
(L/R)TIBIAO	Left/right Tibia	Segment Origin
(L/R)TIBIAA		Anterior axis
(L/R)TIBIAP		Proximal axis
(L/R)TIBIAL		Lateral axis
(L/R)FOOTO	Left/right Foot	Segment Origin
(L/R)FOOTA		Anterior axis
(L/R)FOOTP		Proximal axis
(L/R)FOOTL		Lateral axis
(L/R)TOESO	Left/right Toe	Segment Origin
(L/R)TOESA		Anterior axis
(L/R)TOESP		Proximal axis
(L/R)TOESL		Lateral axis
(L/R)HJC	—	Left/right Hip Joint Center
(L/R)KJC	—	Left/right Knee Joint Center
(L/R)AJC	—	Left/right Ankle Joint Center
(L/R)FJC	—	Left/right Foot Joint Center
(L/R)SJC	—	Left/right Shoulder Joint Center
(L/R)EJC	—	Left/right Elbow Joint Center
CentreOfMass	—	Center of Mass

Segment coordinate system definition was presented on the Plug-In-Gait reference guide (available on the Vicon website - https://www.vicon.com/). Dimensions of trajectories are 3 × N. Units are mm.

**Table 4:** Kinematics and kinetics of the Conventional Gait Model stored in post-processed files.

Labels	Units	Description
(L/R)HipAngles	(deg, deg, deg, ms)	Left/right hip angles
(L/R)KneeAngles	(deg, deg, deg, ms)	Left/right knee angles
(L/R)AnkleAngles	(deg, deg, deg, ms)	Left/right ankle angles
(L/R)ForeFootAngles	(deg, deg, deg, ms)	Left/right forefoot angles
(L/R)SpineAngles	(deg, deg, deg, ms)	Left/right spine angles
(L/R)ShoulderAngles	(deg, deg, deg, ms)	Left/right shoulder angles
(L/R)ElbowAngles	(deg, deg, deg, ms)	Left/right elbow angles
(L/R)WristAngles	(deg, deg, deg, ms)	Left/right wrist angles
(L/R)NeckAngles	(deg, deg, deg, ms)	Left/right neck angles
(L/R)FootProgressAngles	(deg, deg, deg, ms)	Left/right foot progression angles
(L/R)PelvisAngles	(deg, deg, deg, ms)	Left/right pelvis angles
(L/R)ThoraxAngles	(deg, deg, deg, ms)	Left/right thorax angles
(L/R)HeadAngles	(deg, deg, deg, ms)	Left/right head angles
(L/R)GroundReactionForce	(N/kg, N/kg, N/kg, ms)	Left/right ground reaction force
(L/R)HipForce	(N/kg, N/kg, N/kg, ms)	Left/right hip force
(L/R)KneeForce	(N/kg, N/kg, N/kg, ms)	Left/right knee force
(L/R)AnkleForce	(N/kg, N/kg, N/kg, ms)	Left/right ankle force
(L/R)GroundReactionMoment	(N.mm/kg, N.mm/kg, N.mm/kg, ms)	Left/right ground reaction moment
(L/R)HipMoment	(N.mm/kg, N.mm/kg, N.mm/kg, ms)	Left/right hip moment
(L/R)KneeMoment	(N.mm/kg, N.mm/kg, N.mm/kg, ms)	Left/right knee moment
(L/R)AnkleMoment	(N.mm/kg, N.mm/kg, N.mm/kg, ms)	Left/right ankle moment
(L/R)HipPower	(W/kg, ms)	Left/right hip power
(L/R)KneePower	(W/kg, ms)	Left/right knee power
(L/R)AnklePower	(W/kg, ms)	Left/right ankle power

### Missing data

In order to check platform steps, video was only available for overground walk and run. Additionally, no videos were recorded for subject GM001 for all trials during the first session, and for subject HN021 for walking tasks during the first session.

## Data Records

### C3D and CSV files

All data records are available from figshare^30^. They are all stored in c3d file format (https://www.c3d.org). This file format is a public binary file format supported by all motion capture system manufacturers and biomechanics software programs. It is commonly used to store, for a single trial, synchronized 3D markers coordinates and analog data as well as a set of metadata (e.g. measurement units, custom parameters specific to the manufacturer software application). Additionally, data are also stored in CSV files. This file format can be read with any text data reading tools. Trial files are referenced in our dataset in hierarchical folders ID/Session/Trial/Speed/Data/Files.extention with:

- ID (Folder): unique identifier for the volunteer
- Session (Folder): the session (Session1 or Session2)
- Trial (Folder): overground walking (Overground_Walk), treadmill walking (Treadmill_Walk), overground running (Overground_Run), treadmill running (Treadmill_Run) or Static record (Calibration)
- Speed (Folder): Slow, Comfortable, and Fast (for walk) or, Comfortable and Fast (for run)
- Data (Folder): post-process c3d/CSV file (Post_Process), raw c3d/CSV file (Raw) or videoshow compressed AVI files (Video).

For all of the 30 participants and speeds, a total of 4840 and 18159 gait cycles were measured for overground and treadmill walking, respectively. Moreover, 2931 and 18945 cycles were measured for overground and treadmill running, respectively.

## Technical Validation

### Calibration of the optoelectronic system

The optoelectronic system was calibrated before each session following the instructions available by Vicon’s documentation. For all calibration, residuals (i.e. average of the different residuals of the 2D marker rays that belongs to the same 3D point) were below 0.20 (Arbitrary Units of Vicon), and the standard deviation of the reconstructed wand (i.e. calibration tool) length remained below 1.5 mm (less than 1% of the wand length).

### 3D trajectories of cutaneous reflective markers

For raw data, 3D trajectories of cutaneous reflective markers were not reconstructed and gaps may be present. For post-process data, 3D trajectories of cutaneous reflective markers were fully reconstructed without gap using pattern fill, spline fill or rigid body.

### Comparison with published reference dataset

**Fig. 3:**
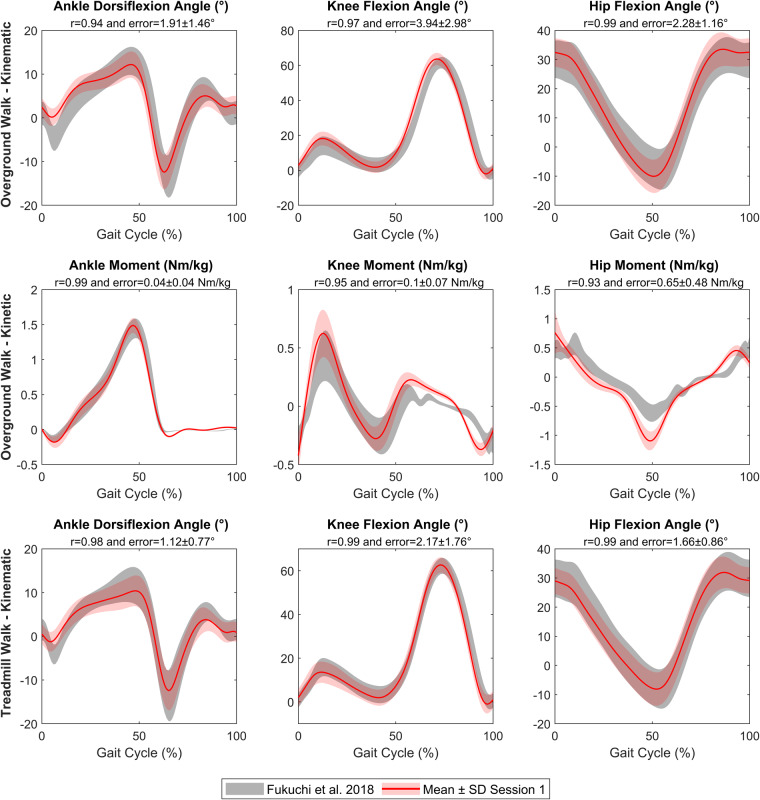
Validation of lower limb kinematic and kinetic during overground and treadmill walk for Session 1 (red). Fukuchi *et al*.^15^ data set was defined as reference (grey). r = Pearson’s correlation coefficient. Error = Mean difference ± standard deviation (SD) between mean curves of each dataset.

**Fig. 4:**
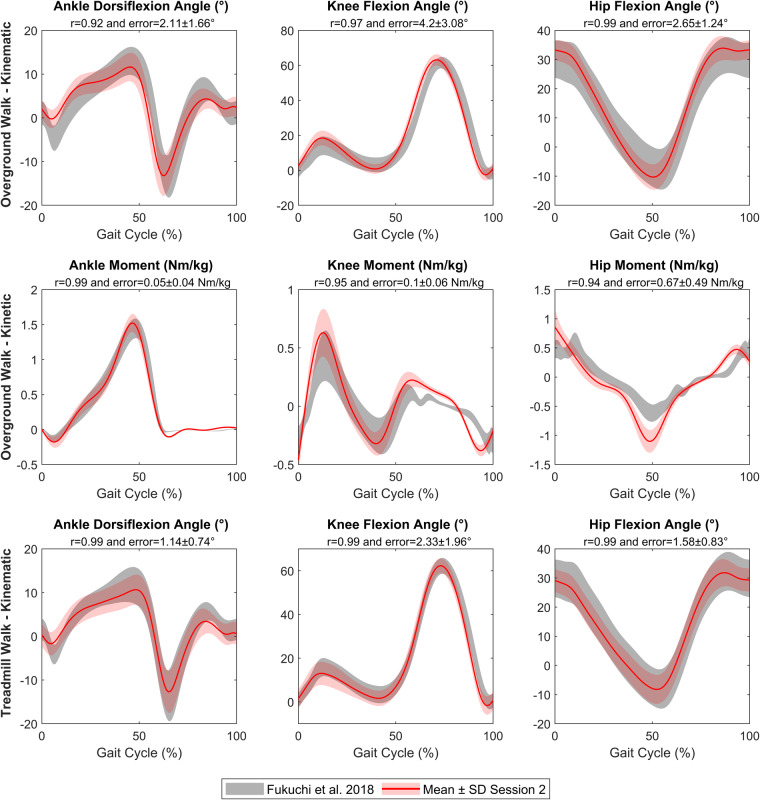
Validation of lower limb kinematic and kinetic during overground and treadmill walk for Session 2 (red). Fukuchi *et al*.^15^ data set was defined as reference (grey). r = Pearson’s correlation coefficient. Error = Mean difference ± standard deviation (SD) between mean curves of each dataset.

Additionally, during walking the movement of the lower body segments occurs mainly in the sagittal plane and can gives a lot of information for gait pathologies^31^. Thus, a validation step was performed for lower limb kinematic and kinetic in the sagittal plane during comfortable walking task, as performed in other datasets^32–34^. Based on overground and treadmill walking acquisition at comfortable speed and using a marker set presented by Leardini *et al*.^35^, the data set published by Fukuchi *et al*.^15^ was used as the reference. This dataset was in accordance with anthropometric parameters of this present study: 24 young adults with age 27.6 ± 4.4 years, height 1.71 ± 1.1 cm, and mass 68.4 ± 12.2 kg. Pearson’s correlation coefficient (r) between the average curves calculated using all gait cycles per session (Session 1 vs Fukuchi *et al*. and Session 2 vs Fukuchi *et al*.) of the two datasets was calculated, as proposed by Ferrari *et al*.^36^. Whatever the session, results highlight excellent correlation between our data (Figs. 3, 4): r values ranged from 0.92 and 0.99 for overground walk (kinematic and kinetic) and from 0.98 and 0.99 for treadmill walk (kinematic). Additionally, for kinematics and kinetics, absolute mean error (±standard deviation) was calculated based on the mean curves of each session compared to Fukuchi *et al*. data (Figs. 3, 4). The median error was 2.1° (CI95% [1.7,2.8]) and 0.10 Nm/kg (CI95% [0.03,0.51]), respectively for kinematic and kinetic mean curves and was in accordance with errors measured in literature^37,38^. Moreover, despite high correlations, some kinematics and kinetic curves were partially outside of the reference range and shifted. These results could be explained by the use of shoes for participants of this present dataset (compared with no shoes for Fukuchi *et al*. participants). Moreover, a speed difference of 0.16 m/s was observed between the two datasets (1.42 m/s and 1.26 m/s, respectively for the present dataset and Fuckuchi *et al*. dataset) that is known to modify gait pattern^39^.

### Limitations

Firstly, the dataset is constrained by a relatively young population (from 21 to 41). Additionally, the sport level of each participant was not evaluated (e.g using questionnaires) which could complicate the comparison in a sportive context. Secondly, the spontaneous speed during overground walk/run could be different during treadmill walk/run which may restrict the comparison between these two tasks.

## Usage Notes

The recorded data are stored in c3d file format (https://www.c3d.org) and can easily be read using c3d toolboxes such as BTK (http://biomechanical-toolkit.github.io/)^40^. The Motion kinematic and kinetic analyzer (Mokka) software can be a convenient tool for 3D visualisation (http://biomechanical-toolkit.github.io/mokka/index.html). Additionally, the recorded data are also stored in CSV file format and can easily be read using any text data reading tools as Excel. Anthropometric and demographic parameters of each participant are stored in the metadata of the related post-process c3d/CSV files and in Excel file. Video can be visualised using VLC software (www.videolan.org).

## Data Availability

A custom Python code used to read data is freely available on the dataset (Python Folder). All processing code used by Vicon are available for free on Vicon website (https://www.vicon.com/).
